# LincRNAs and snoRNAs in Breast Cancer Cell Metastasis: The Unknown Players

**DOI:** 10.3390/cancers14184528

**Published:** 2022-09-19

**Authors:** Maria Louca, Vasiliki Gkretsi

**Affiliations:** 1 Department of Biological Sciences, University of Cyprus, Nicosia 2109, Cyprus; 2 Cancer Metastasis and Adhesion Laboratory, Basic and Translational Cancer Research Center (BTCRC), Nicosia 2404, Cyprus; 3 Biomedical Sciences Program, Department of Life Sciences, School of Sciences, European University Cyprus, Nicosia 2404, Cyprus

**Keywords:** lncRNAs, lincRNAs, snoRNAs, ceRNAs, breast cancer, metastasis

## Abstract

**Simple Summary:**

Recent advances in research have led to earlier diagnosis and more targeted therapies against breast cancer, which has reduced breast cancer related mortality. However, the majority of breast cancer-related deaths are due to metastasis of cancer cells to other sites of the body, a process that has not been fully elucidated. Among the many factors and genes implicated in the regulation of the metastatic process, RNAs that do not code for a specific protein (non-coding RNAs) are emerging as crucial players. This review focuses on the role of long intergenic noncoding RNAs (lincRNAs) and small nucleolar RNAs (snoRNAs) in breast cancer cell metastasis. Both are subclasses of non-coding RNAs that function as regulatory molecules in processes such as cell proliferation, apoptosis, epithelial-to-mesenchymal transition, migration, and invasion, all of which are severely disrupted in aggressive cancer cells that are able to form metastasis in distal sites. In the current review, we identify the most important lincRNAs and snoRNAs that have been recently associated with breast cancer metastasis both through in vitro and in vivo experiments.

**Abstract:**

Recent advances in research have led to earlier diagnosis and targeted therapies against breast cancer, which has resulted in reduced breast cancer-related mortality. However, the majority of breast cancer-related deaths are due to metastasis of cancer cells to other organs, a process that has not been fully elucidated. Among the factors and genes implicated in the metastatic process regulation, non-coding RNAs have emerged as crucial players. This review focuses on the role of long intergenic noncoding RNAs (lincRNAs) and small nucleolar RNAs (snoRNAs) in breast cancer cell metastasis. LincRNAs are transcribed between two protein-coding genes and are longer than 200 nucleotides, they do not code for a specific protein but function as regulatory molecules in processes such as cell proliferation, apoptosis, epithelial-to-mesenchymal transition, migration, and invasion while most of them are highly elevated in breast cancer tissues and seem to function as competing endogenous RNAs (ceRNAs) inhibiting relevant miRNAs that specifically target vital metastasis-related genes. Similarly, snoRNAs are 60–300 nucleotides long and are found in the nucleolus being responsible for the post-transcriptional modification of ribosomal and spliceosomal RNAs. Most snoRNAs are hosted inside intron sequences of protein-coding and non-protein-coding genes, and they also regulate metastasis-related genes affecting related cellular properties.

## 1. Introduction

### 1.1. Breast Cancer Metastasis

Breast cancer has been characterized as the most commonly diagnosed cancer in women, according to Global Cancer statistics 2020 [1] with an estimated 2.3 million new cases (11.7%) and 6.9% mortality rate. In fact, recent advances in diagnostic approaches have led to an earlier diagnosis, which coupled with novel targeted therapeutics, has resulted in a significant decrease in breast cancer-related mortality. However, although, all the above are quite encouraging, breast cancer remains an important health issue that affects the lives of thousands of women across the globe. More importantly, the primary tumor is seldom responsible for cancer-related deaths which are mainly the result of breast cancer metastasis [2].

Current therapeutic approaches target many different signaling pathways that inhibit cancer cell proliferation, induce apoptosis, or interfere with DNA synthesis and repair. Similarly, cancer research is being conducted in various fields of cancer biology. However, little is known regarding the exact molecular mechanism that leads to metastasis, the process of cancer cell spreading in distant parts of the body, and subsequently how this knowledge can be exploited therapeutically. 

**Figure 1:**
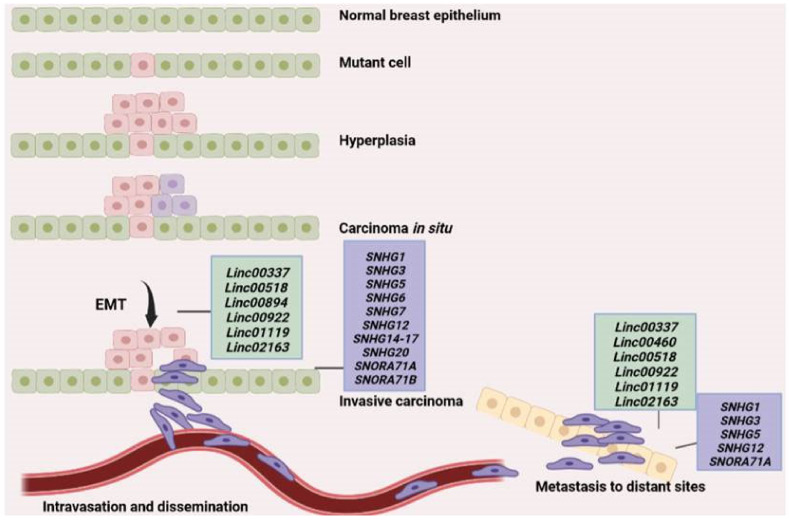
Schematic representation of the metastatic process. A mutation in one of the cells of the normal breast epithelium leads to hyperplasia, while accumulation of additional mutations leads to tranformation of cells into cancer cells and the generation of a tumor that is initially confined to the primary site (carcinoma in situ). Upon activation of various molecular pathways, cancer cells undergo EMT and some of them eventually migrate and invade surrounding tissues or intravasate into neighboring blood vessels through which they are able to reach distant organs or parts of the body where they can extravasate, adhere and establish a new metastatic tumor in the new site. The long intergenic noncoding RNAs (lincRNAs) and small nucleolar RNA (snoRNA) found to be involved in the major steps of the metastatic process are shown in green and purple boxes, respectively.

Metastasis is a complex process comprising multiple stages that ultimately lead to the spreading of cancer cells to other sites of the body that can be close or distant to the primary tumor site (Figure 1). Initially, cancer cells acquire mutations that enable them to lose control of the cell cycle leading to aberrant prolilferation and hyperplasia. Accumulation of additional mutations results in the transformation of cells into cancer cells that express a different set of genes compared to normal cells and have the capacity to grow uncontrollably at the primary site. This is considered as carcinoma *in situ*. Gradually cancer cells lose contact with one another and thus dissociate from their adhesion to neighboring cells and the extracellular matrix while changes in gene expression facilitate the transition of cancer cells from an epithelial phenotype characterized by close connection between cells to a more mesenchymal-like phenotype where cells are loosely connected to each other and acquire a spindle-shaped structure, a process widely known as epithelial-to-mesencymal transition (EMT) [3]. A variety of genes and molecular pathways have been associated with EMT including Wnt-β-catenin pathway, transforming growth factor beta (TGF-β), and Notch-1. This activation of various molecular pathways during EMT leads to activation of genes that control the cytoskeleton such as Rho GTPases, Rac, Rho, and Cdc42, as well as Rho associated coiled-coil containing protein kinase (ROCK) which results in actin cytoskeleton reorganization and concomitant formation of membrane protrusions that are essential for cell migration such as lamellipodia, filopodia, and invadopodia [2]. This is a dramatic change for cancer cells as it enables them not only to migrate but also to activate a set of proteases and matrix metalloproteinases (MMPs) to degrade surrouding extracellular matrix and invade into adjacent tissues. Often, invasion into neighboring blood (intravasation) or lymphatic vessels will facilitate the spreading of cancer cells through circulation or the lymphatic system to other distant organs, where they extravasate, and establish a new colony of malignant cells at the new site. A schematic description of the metastatic process is shown in Figure 1.

Interestingly, apart from various protein-coding genes that have been found to be deregulated in breast cancer metastasis, emerging data suggest that an equally pivotal role is played by non-coding RNAs (ncRNAs) [4].

### 1.2. Noncoding RNAs (ncRNAs)

Even though research tends to focus mainly on protein coding regions of the DNA, recent literature suggests that 99% of the total cellular RNA content actually consists of non-coding RNAs (ncRNAs).

The majority of these ncRNAs are the transfer RNA (tRNA) (89%) and ribosomal RNA (rRNA) (8.9%) followed by messenger RNAs (mRNAs) (0.9%) [5]. The remaining small amount of ncRNAs includes a variety of different ncRNA species such as circular RNA (circRNA), small nuclear RNA (snRNA), small nucleolar RNA (**snoRNA**), miRNA, and long non-coding RNA (**lncRNA**). Although found in such small amounts within the cell, these ncRNAs play critical roles in transcription, post-transcriptional modifications, and translation while it has been suggested that the biological function of each one should be analyzed on a case-by-case basis [5,6].

### 1.3. Long Non-Coding RNAs (lncRNAs)

LncRNAs are defined as RNAs whose transcript length exceeds 200 nucleotides and is not translated into proteins. They are transcribed by RNA polymerase II [5] and although they can be categorized based on their length, function, location, and mechanism of action, so far there are no standard guidelines for this classification [7]. 

According to their position in the genome in relation to protein-coding genes, they are categorized as (i) sense, which are transcribed in the same direction as that of the protein-coding gene, (ii) antisense, which are transcribed in a direction opposite to that of the protein-coding gene, (iii) bidirectional, which are transcribed in both directions, (iv) intronic, that are transcribed from introns found within protein-coding genes, (v) intergenic (lincRNAs), that are transcribed between two protein-coding genes, and (vi) enhancer lncRNAs [5].

The mechanism by which lncRNAs regulate gene expression is complex and greatly depends on their position. They can, therefore, act by being directly bound to DNA or transcription factors thus regulating gene expression at the transcriptional level, they can target mRNAs, miRNAs, or proteins regulating their activities post-transcriptionally, but they can also act epigenetically, by modifying chromatin to activate or inhibit the expression of genes [7].

In general, lncRNAs are highly heterogeneous in terms of structures, being able to take different 3D shapes and conformations which allows them to interact with a variety of macromolecules [8] and finally modulate fundamental biological processes such as development and differentiation, epigenetics, mRNA processing, and protein stability. Interestingly, lncRNAs can also serve as ‘sponges’ (or competing endogenous RNAs-ceRNAs) for miRNAs, thus abolishing the repressive activity of specific miRNAs [5]. To date, over 10,000 lncRNAs have been identified in the human genome and their number is still rising.

### 1.4. LncRNAs in Cancer

Based on the many different levels upon which lncRNAs act, it is evident that their function is essential for maintaining normal homeostasis and their deregulation is linked to various human diseases [9,10]. Notably, a large number of studies have shown that lncRNAs are frequently deregulated in cancer while an association has been revealed between lncRNA deregulation, oncogenicity, and cancer progression [11,12,13,14,15]. More specifically, lncRNAs have been shown to regulate multiple cancer hallmarks such as viability, proliferation, migration, angiogenesis, and cellular immortality [16], while many studies have indicated that lncRNAs have cancer type-specific deregulation patterns [17,18,19]. Hence, it has been suggested that lncRNAs have the potential of being attractive anti-cancer therapeutic targets [20]. 

This article focuses on the review of the current literature on the role of two less known types of lncRNAs, namely long intergenic noncoding RNAs (lincRNAs) and small nucleolar RNA (snoRNAs) in breast cancer cell metastasis.

### 1.5. Long Intergenic Noncoding RNAs (LincRNAs)

**Figure 2:**
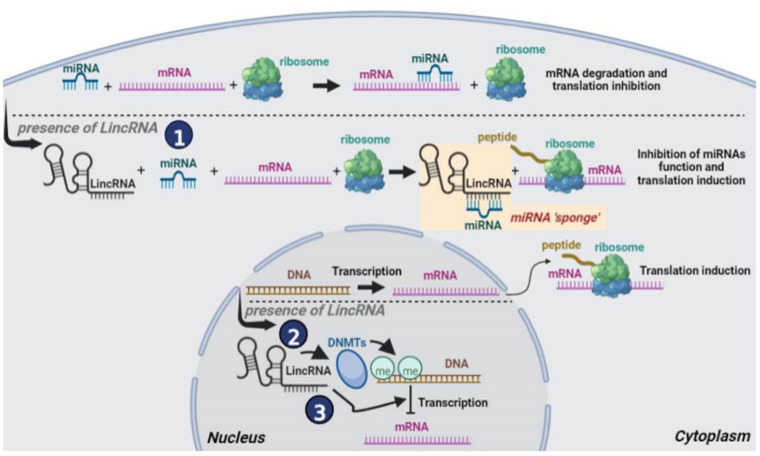
Schematic representation of the function of lincRNAs in breast cancer cells: (1) In the cytoplasm, lincRNAs sponge specific miRNAs repressing their functions and thus repressing specific mRNA translation. The sponging process is shown inside the yellow box. (2) In the nucleus, lincRNAs attract DNMTs to specific gene promoters whose methylation represses gene expression, and (3) In the nucleus lincRNAs inhibit the expression of tumor suppressor genes.

Long intergenic noncoding RNAs (lincRNAs), that are transcribed between two protein-coding genes, belong to the broader category of lncRNAs and also consist of transcripts longer than 200 nucleotides. As lncRNAs they also do not code for a specific protein but rather function as regulatory molecules playing a crucial role in a variety of cellular processes, including gene expression regulation and splicing. Moreover, their functions are characterized by cell-type specific expression and subcellular compartment localization [21,22] while their deregulation is also associated with breast cancer development and progression through their interaction with cancer related genes [23,24,25]. LincRNAs can act in various ways inside the cell [5]. Inside the cytoplasm, lincRNAs serve as “sponges” for specific miRNAs thus leading to the repression of the specific mRNAs that the miRNAs target (Figure 2, route 1). Inside the nucleus, lincRNAs either recruit DNA methyltransferases (DNMTs) to specific gene promoters leading thus to inhibition of the expression of the respective genes (Figure 2, route 2) or they directly inhibit the expression of tumor suppressor genes (Figure 2, route 3).

Several lincRNAs have been recently identified as key players in breast cancer pathogenesis and metastasis.

***(A) Linc00337:*** Linc00337 is located on chromosome 1 and it has been implicated in breast, lung, gastric, colorectal, and esophageal squamous cell carcinoma progression via various mechanisms [26,27,28,29,30]. Subcellular localization analysis revealed that it is mainly localized in the nucleus being implicated in epigenetic regulation of gene expression [26,30]. In breast cancer, linc00337 levels are elevated compared to adjacent normal breast tissues [26]. Moreover, linc00337 silencing decreases the viability and cancer cell proliferation, while Linc00337 overexpression has the opposite effect [26]. Further in vitro and in vivo analysis revealed that Linc00337 not only enhances the malignant phenotype of breast cancer cells but also promotes chemoresistance to the chemotherapeutic drug paclitaxel [31] through M2-like macrophages [26], which are considered to interact with tumor cells and promote tumor development [32,33,34]. Specifically, Linc00337 was found to upregulate the expression of M2 tumor-associated macrophage markers promoting cell migration and EMT as well as resistance to paclitaxel, a known chemotherapeutic agent used in breast cancer treatment.

***(B) Linc00460:*** Linc00460 is located on the 13 chromosome and is 935 bp in length [35]. It has recently been reported to be involved in the regulation of the malignant phenotype of multiple cancers including breast, lung, colorectal, prostate, colon, and ovarian cancer and it has been proposed as a potential novel diagnostic and therapeutic target [36,37,38,39,40,41,42]. Moreover, Linc00460 has been found to serve as a ceRNA targeting a number of tumor suppressor miRNAs and finally leading to oncogenesis [37]. Specifically in breast cancer, linc00460 was shown to serve as a ceRNA for Fibroblast Growth Factor 7 (FGF-7) mRNA by sponging miR-489-5p leading to upregulation of FGF7 expression and AKT activity and ultimately oncogenesis [39]. Further to this, high levels of Linc00460 are correlated with shorter overall survival in breast cancer patients [39] while its silencing prevented tumor growth in in vivo experiments and attenuated the malignant progression of breast cancer cells. Consistent with these finding, Linc00460 overexpression enhanced tumor growth and malignant progression [39].

***(C) Linc00518:*** Linc00518 is located on human chromosome 6p24.3 and shows relatively low expression in most normal human tissues [43]. However, it is highly expressed in various types of cancer such as melanoma and breast cancer and it is thought to be involved in cancer progression [43,44]. Specifically, regarding breast cancer, it was recently shown that overexpression of Linc00518 in MCF-7 breast cancer cells recruited DNMT on caudal type homeobox 2 (CDX2) gene promoter and its methylation decreased the CDX2 expression leading to activation of the Wnt signaling pathway with accompanying upregulation of β-catenin, c-Myc, CyclinD1, Slug, Snail, Twist, and downregulation of E-cadherin [45], clearly showing promotion of EMT. Linc00518 silencing on the other hand further validated the overexpression findings as it resulted in decreased proliferation, migration, and invasion, enhanced apoptosis and inactivation of Wnt signaling pathway, indicating reduced tendency to EMT [45]. Moreover, Linc00518 silencing enhanced the antitumor effect of the chemotherapeutic agent doxorubicin and promoted cancer cell apoptosis, suggesting that Linc00518 increases resistance to chemotherapy [44].

These findings were verified in vivo in nude mice injected with MCF-7 cells that had been previously transfected with short hairpin RNA (shRNA-Linc00518). Tumors grown from these cells displayed reduced growth and metastasis rates, suggesting that Linc00518 by itself promotes tumorigenesis and metastasis. Finally, Linc00518 serves as molecular sponge of miR-199a and inhibits multidrug resistance-associated protein (MRP1) expression which increases the sensitivity of breast cancer cells to doxorubicin and paclitaxel [44].

***(D) Linc00641:*** LINC00641 is a novel lncRNA located on chromosome 14, locus 14q11.2 [46]. Recent studies have demonstrated that Linc00641 can serve as a ceRNA via sponging specific miRNAs thus inhibiting the malignant progression of many cancer types including breast, glioma, bladder, lung, and cervical cancer [47,48,49,50,51]. In breast cancer, Linc00641 was shown to interact and inhibit miR-194-5p, whose expression is higher in breast cancer tissues compared to normal and is thought to promote breast cancer growth and metastasis by modulating the Wnt/β-catenin signaling cascade acting as oncogene [52]. The use of bioinformatic databases such as TANRIC and GEPIA also revealed that Linc00461 levels are diminished in human breast cancer tissues compared to normal-adjacent tissues [49] and are negatively correlated to tumor size, lymph node metastasis, and clinical stages showing that Linc00641 functions as tumor suppressor in breast cancer. In fact, in vitro overexpression of Linc00641 showed inhibition of breast cancer proliferation by preventing transition to the G1/S phase of the cell cycle, inhibition of migration and invasion in many cancer types, and induction of apoptosis. Moreover, in vivo studies revealed that Linc00641 overexpression notably reduces the tumor growth and the metastasis of breast cancer cells [49].

***(E) Linc00894:*** Linc00894 is a lncRNA derived from X chromosome and it was found to be upregulated in breast cancer tissues compared to adjacent normal ones as well as in the AU565 aggressive breast cancer cell line compared to the less aggressive MCF-7 cells [53]. Additionally, Linc00894 silencing resulted in reduced proliferation of breast cancer cells, and reduced invasion capacity while its overexpression had the exact opposite results enhancing the metastatic properties of breast cancer cells. These results were also validated in vivo using the xenograft model in mice [53]. Specifically, elimination of Linc00894 reduced the volume and the weight of breast tumors and also impaired lung metastasis compared to control tumors [53]. In addition, breast cancer patients with high Linc00894 expression had shorter overall survival compared to those with low expression indicating that Linc00894 promotes cancer progression [53]. Again, Linc00894, similarly to Linc02163 and Linc01977 functions at the post-transcriptional level as a ceRNA. In fact, miR-429 was shown via bioinformatic analysis to be regulated by Linc00894. In brief, Linc00894 was found to competitively bind to miR-429 and regulate the expression of transcriptional factor zinc finger E-box binding homeobox 1 (ZEB1) which leads to breast cancer progression [53].

***(F) Linc00922:*** Linc00922 is located on chromosome 16 and was recently implicated in cancer initiation and progression of breast, ovarian, lung, and liver cancer [54,55,56,57,58]. Wang et al., in 2021 investigated in detail the role of Linc00922 in breast cancer progression [55]. Using the microarray dataset GSE26910 and the GEO database they revealed that Linc00922 expression is elevated in breast cancer tissues compared to its adjacent counterparts. Furthermore, they observed that ectopic expression of Linc00922 activated the Wnt signaling pathway that is known to be activated in breast cancer and is correlated with poor prognosis in breast cancer patients promoting EMT, cell proliferation, migration, and invasion, as well as tumor growth and metastasis in vivo [59]. Fluorescent in situ hybridization (FISH) analysis revealed that Linc00922 is mainly localized in the nucleus of MCF-7 breast cancer cells where it acts as an oncogene recruiting three different DNMTS; DNMT1, DNMT3A, and DNMT3B in the promoter region of NKD inhibitor of Wnt signaling pathway 2 (NKD2), thus epigenetically regulating NKD2 expression. This is indeed critical, as NKD2 is often methylated and poorly expressed in breast cancer while it also antagonizes the Wnt signaling pathway, suppressing tumor growth and metastasis [60,61,62]. In support of the above-described findings, elimination of Linc00922 led to opposite results [55]. In fact, all in vitro data were validated in vivo. First, experiments using ectopic expression of Linc00922 revealed that it increases tumor growth in xenograft mouse models of breast cancer through inhibition of NKD2, thereby enhancing breast cancer metastasis to the lung and the liver [55].

***(G) Linc01087:*** LINC01087 is another newly discovered lncRNA which is located on 2q21.1 chromosome and has been shown to play a crucial role in glioma and breast cancer [63,64]. Contrary to all previously-discussed lincRNAs, RNA-sequencing data from breast cancer patients revealed that Linc01087 is significantly downregulated in patients with triple negative breast cancer (TNBC), who have worse prognosis and high risk of recurrence and metastasis, compared to samples from cancer-free women while it is upregulated in patients with luminal breast cancer, who usually have better prognosis [65,66]. In addition, Kaplan–Meier plotter analysis showed that TNBC patients with low Linc011087 levels displayed positive lymph node status but patients with luminal breast cancer and high Linc01087 expression had extended relapse free survival (RFS) [67]. Thus, it is obvious that the low levels of Linc01087 are associated with disease progression of BC patients while high levels seem to have a protective effect.

***(H) Linc01119:*** Linc01119 is located on chromosome 2, locus 2p21 and has been implicated in the initiation and progression of colorectal and breast cancer [68,69]. A recent study specifically showed that inhibition of Linc01119 using specific inhibitor led to significant reduction in the cell growth of Hs578T, MDA-MB-468, and CAL51 TNBC cell lines in vitro as well as reduction of tumor growth in vivo [68]. Moreover, Linc01119 was found to stimulate the expression of the suppressor of cytokine signaling 5 (SOCS5) which inhibits the Janus activated kinase (JAK1/2) and the phosphorylation of signal transducer and activator of transcription (STAT1/3) pathway, thus promoting breast cancer cell growth in vitro and tumorigenesis in vivo [68,69,70,71]. Additionally, Kaplan–Meier plot analysis revealed an association between high Linc01119 and SOCS5 expression levels and shorter RFS in breast cancer patients [68].

***(I) Linc01977:*** Linc01977 is also a newly described lncRNA located on chromosome 17, locus 17q25.3, has a length of 1799bp and was recently proposed as a prognostic marker for lung and breast cancer [72,73]. Moreover, Linc01977 silencing in MDA-MB-231 and MCF-7 breast cancer cells impaired breast cancer cell growth and motility and also rendered cells more sensitive to the chemotherapeutic drug doxorubicin (DOX) [73]. Notably, and in accordance with these findings, bioinformatic analysis using The Cancer Genome Atlas (TCGA) and GSE155478 datasets revealed that Linc01977 expression is elevated in breast cancer cell lines with DOX resistance while breast cancer patients with remarkably high levels of Linc01977 have significantly lower overall survival compared to those with lower Linc01977 levels [73]. Similar to Linc02163, Linc01977 is also mainly localized in the cytoplasm and functions as a ceRNA by sponging, or specifically binding, miR-212-3p and by increasing the levels of its target gene Golgi membrane protein 1 (GOLM1) that encodes a Golgi associated protein and promotes an oncogenic phenotype in many cancer types [74,75].

***(J) Linc02163:*** Among other lincRNAs found to be deregulated in cancer, Linc02163 was recently shown to play a role in colorectal and breast cancer [76,77]. Linc02163 is located on chromosome 5q21.2 and it was found to be upregulated in breast cancer tissues compared to adjacent normal tissues with the findings being verified in four different breast cancer cell lines (SKBR3, MDA-MB-231, MCF-7, and BT-474) as compared to human immortalized breast epithelial cell line MCF-10A [77]. Moreover, Linc02163 silencing resulted in reduced cell proliferation, migration, and invasion of breast cancer cells and increased apoptosis [77]. Interestingly, nuclear/cytoplasmic fractionation revealed that Linc02163 is mostly localized in the cytoplasm where it is suggested to have a post-transcriptional role functioning as a ceRNA for miRNAs and consequently decreasing the regulatory impact of these miRNAs on their target mRNAs [78,79]. For instance, in vivo studies using a xenograft mouse model showed that it can serve as a ceRNA for the miR-511-3p upregulating high-mobility group A2 (HMGA2) gene [77], which is a specific target of miR-511-3p. Furthermore, survival analysis using the Kaplan–Meier plotter revealed that patients with high Linc02163 levels exhibited shorter overall survival, larger tumor size, and greater metastatic potential, indicating that Linc02163 expression is correlated with poor prognosis for breast cancer patients [77].

***(K) Linc02615:*** Last but not least, Linc02615 was very recently discovered but little is known regarding its expression and function. It is located on chromosome 4, locus 4q28.2 and has a length of 742 bases. Similarly to Linc01087 and contrary to all previously described lincRNAs, its expression is lower in breast cancer tissues compared to healthy ones and it is therefore postulated to function as a tumor suppressor [80], based on data extracted from the CoLncRNA database using a bioinformatic approach [80]. Finally, Linc02615 also acts as a ceRNA targeting the miR-129-5p and consequently upregulates the expression of its target genes such as lamins [81]. A more detailed investigation on the exact role and mechanism of action of Linc02615 in breast cancer is thus needed.

A summary of the studies that investigate the connection between various lincRNAs and breast cancer progression and metastasis is given in Table 1.

### 1.6. Small Nucleolar RNAs (snoRNAs)

Small nucleolar RNAs (snoRNAs) are 60–300 nucleotides in length and as the name suggests, are mainly found in the nucleolus where they function as guide RNAs for the post-transcriptional modification of ribosomal RNAs and some spliceosomal RNAs [4].

**Figure 3:**
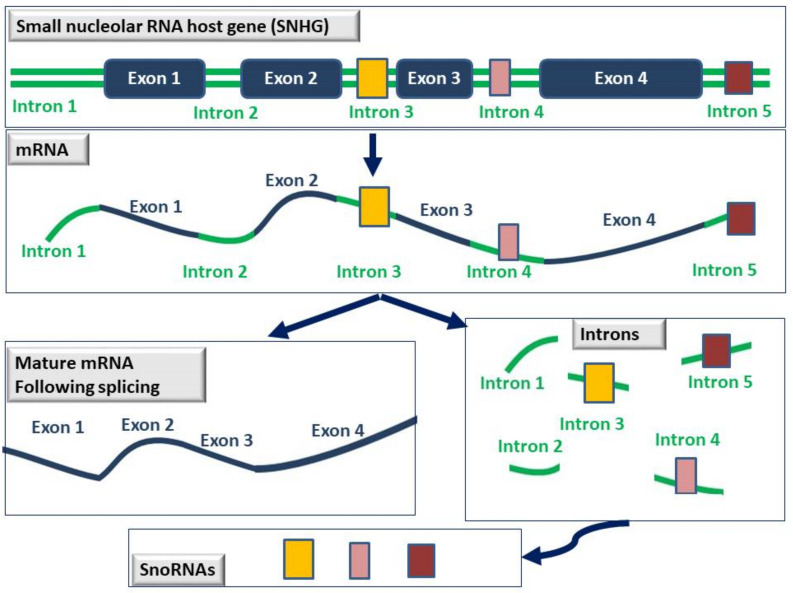
Schematic representation of the process by which snoRNAs are generated. SnoRNAs (depicted as yellow, pink, or brown boxes) are usually hosted within the intron sequences of genes known as small nucleolar RNA host genes (SNHGs). Following splicing, exons are connected together to generate the mature mRNA, and the introns are further processed into snoRNAs that are now fully functional in the nucleolus.

The vast majority of snoRNAs are hosted inside the intron sequences of protein-coding and non-protein-coding genes, termed small nucleolar RNA host genes (SNHGs). During splicing, introns are removed and exons are connected together to form the mature mRNA. After splicing though, introns are not lost or degraded but are further processed into snoRNAs and function in the nucleolus [4] (see Figure 3 below).

To date, there are 22 members of the SNHG family (SNHG1 to SNHG22) many of which have been shown to be deregulated in cancer, being critically involved in processes such as cancer cell proliferation, tumor progression, metastasis, and chemoresistance [4].

### 1.7. SnoRNAs in Breast Cancer

For instance, in breast cancer, the observed enhancement in protein synthesis is thought to be due to elevated rRNA expression for which elevated snoRNA biogenesis is required [82]. Thus, several snoRNAs have been identified to be implicated in breast cancer pathogenesis and have prognostic value in breast cancer.

SNHG1 was found to be significantly upregulated in human breast cancer tissues and cell lines [83] and shown to promote breast cancer cell proliferation and metastasis both in vitro and in vivo [83]. Regarding the molecular mechanism of action, SNHG1 was shown to act as a sponge for miR-193a-5p finally activating the expression of homeobox A1 (*HOXA1*) oncogene [83] but it was also shown to act through regulation of miR-382 and miR-448 expression [84,85]. Moreover, SNHG1 was upregulated under hypoxic conditions in breast cancer cells MDA-MB-231 in a hypoxia-inducible factor-1 (HIF-1) dependent manner and was found to be co-expressed with miR-199a-3p regulating its target gene transcription factor A mitochondrial (TFAM), ultimately leading to breast cancer cell metastasis [86]. Finally, SNHG1 was found to act as sponge for miR-18b-5p regulating the expression of telomerase reverse transcriptase (TERT). Interestingly, in vivo experiments combining SNHG1 knockdown and TERT inhibition dramatically inhibited breast tumor growth [87].

Similarly, SNHG3 expression was dramatically elevated in breast cancer cells and tissues, while overexpression of SNHG3 in MCF-7 band MDA-MB-231 cells induced breast cancer cell proliferation, EMT, migration, and invasion, through regulating miR-186-5p and ZEB1 expression [88]. Further, SNHG3 silencing led to breast cancer cell growth inhibition both in vitro and in vivo [83] and it was found to act as sponge for miR-154-3p regulating Notch signaling. Lastly, secretion of SNHG3 from breast cancer associated fibroblasts promotes breast cancer cell proliferation via its action as a sponge for miR-330-5p [89] which inhibits pyruvate kinase M1/M2 (PKM) and miR-384 which inhibits hepatoma derived growth factor (HDGF) and also induces breast cancer cell migration, and invasion [90].

SNHG5 acts as a sponge for miR-154-5p, which normally suppresses proliferating cell nuclear antigen (PCNA), and therefore promotes cell cycle progression in breast cancer cells and inhibits apoptosis at the same time [91]. SNHG6 promotes breast cancer cell proliferation, migration, and invasion by acting as a sponge for miR-26a-5p, which inhibits vasodilator-stimulated phosphoprotein (VASP) [92]. SNHG7 has also been connected to breast cancer pathogenesis and metastasis through a number of studies showing that breast cancer cells and tissues express elevated SNHG7 levels that are correlated to tumor stage, distant metastasis, lymph node metastasis, and reduced overall survival [93,94,95]. Specifically, SNHG7 binds to c-Myc oncogene and enhances its expression [96] while it also acts as a sponge for miR-34a [95] as silencing studies showed that it affects the expression of miR-34a and the Notch-1 pathway that it regulates [95]. Furthermore, it acts as a sponge for miR-186 [94] and miR-381 [93], which are also implicated in breast cancer pathogenesis while it was also shown to bind to miR-15a [97].

Similarly, SNHG12 also has a connection with c-Myc as it is a transcriptional target of the oncogene and it is also found upregulated in TNBC where it is correlated with advanced tumor stage and size, and reduced overall survival [98]. In fact, SNHG12 upregulation promotes proliferation, inhibits apoptosis, and induces breast cancer cell migration whereas SNHG12 silencing inhibits breast cancer cell growth in vitro and in vivo [99]. In fact, SNHG12 was shown to interact with and act as sponge for miR-15a-5p to promote Sal-like 4 (SALL4) expression [99].

In accordance with the above, SNHG14 and SNHG15 were also shown to promote cancer cell proliferation, migration, and invasion [100,101,102,103]. Specifically, SNHG14 expression was found elevated in breast cancer cells and tissues and its silencing attenuated cancer cell proliferation, migration, and invasion while at the same time enhancing apoptosis. Notably, overexpression yielded the exact opposite effects [87]. Regarding the molecular mechanism of action, SNHG14 was shown to directly interact as sponge with miR-543 and regulated Krüppel-like factor 7 (KLF7). Furthermore, SNHG14 acts by epigenetically regulating the acetylation of histones including histone H3K27 but it can also act as a sponge for miR-193a-3p [102]. Regarding SNHG15, it is also highly expressed in breast cancer tissues and cell lines and is positively associated with larger tumor size, lymph node metastasis, and decreased survival, while it also acts as a sponge for miR-411-5p [101] and miR-211-3p [103].

Finally, breast cancer cell proliferation, migration, and invasion are also promoted through the action of SNHG16 [104,105], SNHG17 [106], and SNHG20 [107] via sponging miR20a, miR-124-3p, and miR-495, respectively.

Interestingly, through a small RNA sequencing screen for snoRNAs related to EMT in breast cancer, Hu et.al. showed that SNORA71A promotes proliferation, migration, invasion, and EMT in MCF-7 and MDA-MB-231 cells while its overexpression in vivo clearly promotes breast tumor growth, and its knockdown inhibits breast cancer growth and metastasis [108]. From the molecular mechanism point of view, SNORA71A was shown to act through regulation of ROCK2, a negative regulator of TGF-β signaling. Finally, SNORA71B was found significantly elevated in brain metastasis breast cancer tissues compared to respective controls where it was also correlated with poor overall survival in breast cancer patients. Moreover, SNORA71B was shown to induce breast cancer proliferation, EMT, migration, and invasion while its silencing inhibited the high brain metastasis breast cancer cells across the blood–brain barrier [109].

A summary of the studies showing the involvement of snoRNAs in promoting metastatic properties both in vitro and in vivo is given in Table 2.

### 1.8. LincRNAs and snoRNAs as Biomarkers of Disease Progression

It is a fact that several of the lincRNAs, snoRNAs and SNHGs described above (Table 1 and Table 2) are found upregulated in breast cancer samples and this upregulation coincides, in some cases, with reduced survival (Table 1). This, by definition, is an indication that lincRNAs, snoRNAs. and SNHGs could be utilized as diagnostic biomarkers and/or therapeutic targets for breast cancer. In fact, several studies have already demonstrated that circulating lncRNAs are potential biomarkers in multiple types of cancers, including cholangiocarcinoma, non-small-cell lung cancer, hepatocellular carcinoma, and gastric cancer [110,111]. In breast cancer in particular, RNA sequencing data showed that twenty-five (25) lincRNAs were associated with overall survival among two hundred (200) TNBC samples [112] providing evidence that lincRNAs can be used as potential diagnostic biomarkers for TNBC. Moreover, there is evidence that some lncRNAs can affect chemotherapy sensitivity in BC patients (Table 1) and may thus serve as biomarkers not only of prognostic and diagnostic value but also as biomarkers that will be useful in predicting response to chemotherapy [113]. It is evident that the potential of lincRNAs and snoRNAs as biomarkers is immense and this application will definitely provide new insights into the diagnosis and treatment of breast cancer but more research is undoubtedly needed before they can be put in the clinic.

## 2. Conclusions

Recent advances made in research related to the role of lincRNAs and snoRNAs in breast cancer cell metastasis clearly show that these non-coding types of RNA are crucially involved in the regulation of fundamental genes implicated in EMT, cancer cell proliferation, migration, and invasion, all of which are pivotal for the metastatic process. As shown in Figure 1, Linc00337, Linc00518, Linc00894, Linc00922, Linc01119, and Linc02163 are involved in EMT mainly through sponging specific miRNAs that normally inhibit important pathways such as the Wnt/β-catenin molecular pathway (e.g., Linc005518, Linc00922). Thus, the effect of these lincRNAs is activation of the Wnt/β-catenin pathway and consequently EMT. Moreover, in vivo experiments have shown that Linc00337, Linc00460, Linc00518, Linc00922, Linc01119, and Linc02163 induce tumor growth and metastasis in vivo (Table 1 and Figure 1) which suggests that they could be potential targets for developing novel anti-metastatic therapeutics. Similarly, all studied SNHGs are involved in EMT through activation of various athways including Notch-1, but in vivo experiments reveal a more crucial role in tumor growth and metastasis for SNHG1, SNHG3, SNHG5, SNHG12, and SNORA71A (Table 2 and Figure 1).

Last but not least, most lincRNAs and snoRNAs are found in elevated levels in breast cancer tissues compared to normal which clearly indicates that they have the potential of being potent diagnostic or prognostic biomarkers and they should therefore be evaluated as such in future studies. Finally, the fact that most of them also function as ceRNAs inhibiting different miRNA(s) involved in the regulation of expression of various genes highlights the mere complexity of their function. All in all, the present review reveals that both lincRNAs and snoRNAs have the potential to serve as novel biomarkers and/or therapeutic targets against breast cancer cell metastasis but future research will be definitely required to address all unanswered questions related to these molecules and will add more value to the previously understated non-coding DNA regions.

## Figures and Tables

**Table 1:** Summary of studies correlating the function of different lincRNAs in breast cancer progression and metastasis.

	Levels in Breast Cancer Compared to Normal	Survival	ceRNA Function	miRNA Targeted	Pathway	Proliferation, Migration, Invasion & EMT	*In Vivo* Growth & Metastasis	Chemoresistance (Drug)
**Linc00337**	↑				↑ M2 tumor associated macrophage markers	↑	↑	↑ (Paclitaxel)
**Linc00460**	↑	↓	Yes				↑	
**Linc00518**			Yes	miR199	↑Wnt, β-catenin MRP1	↑	↑	↑ (Doxorubicin, Paclitaxel)
**Linc00894**	↑	↓	Yes	miR429	ZEB1	↑		
**Linc00922**	↑				Wnt, NKD2	↑	↑	
**Linc01119**					↑SOCS5, ↓JAK1/2 ↓ STAT1/3	↑	↑	
**Linc01977**	↑	↓	Yes	miR-212-3p	↓GOLM1			↑ (Doxorubicin)
**Linc02163**	↑	↓	Yes	miR-511-3p	↑HMGA1	↑	↑	
**Linc00641**	↓		Yes	miR-194-5p	↓Wnt, β-catenin	↓	↓	
**Linc01087**	↓ In TNBC							
**Linc02615**	↓		Yes	miR-129-5p	↑Lamins			

**Table 2:** Summary of studies performed on the function of different snoRNAs in breast cancer metastasis using both in vitro and in vivo approaches. Gray rows show the snoRNAs that were studied both in vitro and in vivo while white rows show the snoRNAs that were studied in vitro only.

	*Elevated Levels in Breast Cancer Compared to Nornal*	*ceRNA Function*	*miRNA Targeted and/or Pathway Involved*	*Increased Proliferation, Migration, Invasion & EMT* *(In Vitro)*	*In Vivo Growth and Metastasis*
*SNHG1*	√	√	miR-193a-5p → HOXA1 miR-199a-3p → TFAM miR-18b-5p → TERT miR-382 miR-448	√	√
*SNHG3*	√	√	miR-186-5p → ZEB1 miR-154-3p → Notch miR-330-5p → PKM miR-384-5p → HDGF	√	√
*SNHG5*		√	miR-154-5p → PCNA	√	√
*SNHG6*		√	miR-26a-5p → VASP	√	
*SNHG7*	√	√	c-Myc miR-34a → Notch1 miR-186 miR-381 miR-15a	√	
*SNHG12*	√	√	c-Myc miR-15a-5p → SALL4	√	√
*SNHG14*	√	√	miR-543 → KLF7 acetylation of H2K27 miR-193a-3p	√	
*SNHG15*	√	√	miR-411-5p	√	
*SNHG16*		√	miR-211-3p	√	
*SNHG17*		√	miR-20a	√	
*SNHG20*		√	miR-124-3p miR-495	√	
*SNORA71A*				√	√
*SNORA71B*	√		ROCK2	√	
